# Bone turnover prediction in patients with chronic kidney disease (CKD) undergoing hemodialysis using shortened dynamic ^18^F-NaF PET/CT K_i_–Patlak

**DOI:** 10.1038/s41598-024-63476-z

**Published:** 2024-05-31

**Authors:** Viyada Sanoesan, Jeerath Phannajit, Kanaungnit Kingpetch, Thunyaluk Sawatnatee, Benchamat Phromphao, Paweena Susantitaphong, Chanan Sukprakun, Kitiwat Khamwan

**Affiliations:** 1 Medical Physics Program, Department of Radiology, Faculty of Medicine, Chulalongkorn University, Bangkok, 10330 Thailand; 2 Chulalongkorn University Biomedical Imaging Group, Department of Radiology, Faculty of Medicine, Chulalongkorn University, Bangkok, 10330 Thailand; 3 Department of Radiological Technology, Faculty of Sciences, Ramkhamhaeng University, Huamark, Bangkapi, Bangkok, 10240 Thailand; 4 Division of Clinical Epidemiology, Department of Medicine, King Chulalongkorn Memorial Hospital, The Thai Red Cross Society, Bangkok, 10330 Thailand; 5 Division of Nephrology, Department of Medicine, Faculty of Medicine, Chulalongkorn University, Bangkok, 10330 Thailand; 6 Center of Excellence for Metabolic Bone Disease in CKD Patients, Faculty of Medicine, Chulalongkorn University, Bangkok, 10330 Thailand; 7 Division of Nuclear Medicine, Department of Radiology, Faculty of Medicine, Chulalongkorn University, Bangkok, 10330 Thailand; 8 Division of Nuclear Medicine, Department of Radiology, King Chulalongkorn Memorial Hospital, The Thai Red Cross Society, Bangkok, 10330 Thailand

**Keywords:** Chronic kidney disease, Renal osteodystrophy, Bone turnover prediction, Metabolic bone disease, ^18^F-NaF kinetic modeling, K_i_–Patlak, Predictive markers, End-stage renal disease

## Abstract

This study investigated whether K_i_–Patlak derived from a shortened scan time for dynamic ^18^F-NaF PET/CT in chronic kidney disease (CKD) patients undergoing hemodialysis can provide predictive accuracy comparable to that obtained from a longer scan. Twenty-seven patients on chronic hemodialysis, involving a total of 42 scans between December 2021 and August 2023 were recruited. Dynamic ^18^F-NaF PET/CT scans, lasting 60–90 min, were immediately acquired post-injection, covering the mid-twelfth thoracic vertebra to the pelvis region. K_i_–Patlak analysis was performed on bone time–activity curves at 15, 30, 45, 60, and 90 min in the lumbar spine (L1–L4) and both anterior iliac crests. Spearman’s rank correlation (r_s_) and interclass correlation coefficient were used to assess the correlation and agreement of K_i_–Patlak between shortened and standard scan times. Bone-specific alkaline phosphatase (BsAP) and tartrate-resistant acid phosphatase isoform 5b (TRAP5b) were tested for their correlation with individual K_i_–Patlak. Strong correlations and good agreement were observed between K_i_–Patlak values from shortened 30-min scans and longer 60–90-min scans in both lumbar spine (r_s_ = 0.858, *p* < 0.001) and anterior iliac crest regions (r_s_ = 0.850, *p* < 0.001). The correlation between BsAP and K_i_–Patlak in the anterior iliac crests was weak and statistically insignificant. This finding suggests that a proposed shortened dynamic ^18^F-NaF PET/CT scan is effective in assessing bone metabolic flux in CKD patients undergoing hemodialysis, offering a non-invasive alternative approach for bone turnover prediction.

## Introduction

The diagnosis of renal osteodystrophy (ROD) is typically based on bone turnover, mineralization, and volume (TMV system), which can be assessed using bone histomorphometry from biopsy^1^. ROD in patients with chronic kidney disease (CKD) and end-stage renal disease (ESRD) is determined clinically by bone turnover, which is defined as the rate of skeleton remodeling or the rate of bone formation and resorption. Low bone turnover is associated with reduced osteoblast and osteoclast activities, whereas high bone turnover is associated with increased osteoblast and osteoclast activities^2^. Both low and high bone turnover may lead to bone abnormalities and increased fracture risk. Although bone biopsy is considered the gold standard for the specific classification of the different subtypes of ROD, it is an invasive and painful procedure that is limited to a single bone site and requires considerable expertise to interpret specimens. Furthermore, assessing the impact of treatment on bone turnover requires multiple sequential biopsies because a single biopsy is insufficient to record the changes that occur over time^3^.

Bone metabolic flux (K_i_) obtained from fluorine-18-labeled sodium fluoride (^18^F-NaF) acquired using positron emission tomography/computed tomography (PET/CT) imaging is used as an alternative approach for ROD classification^4–6^. K_i_ represents the net regional plasma clearance of ^18^F-NaF and the bone formation rate. The uptake mechanism of ^18^F-NaF relies on the diffusion of ion exchange from the plasma into the extracellular fluid space within bones and is deposited on the surface of newly formed hydroxyapatite crystals at sites of bone formation, with increasing ^18^F-NaF uptake in locations where osteoblasts and osteoclasts are activated. ^18^F-NaF is rapidly excreted by the kidneys^7–9^. Several studies have shown that K_i_ is positively and significantly correlated with bone turnover parameters obtained using bone biopsies^5,9^. Although K_i_ can serve as an imaging biomarker for assessing regional bone metabolism at one or multiple skeletal sites with a single injection, obtaining the K_i_ value traditionally requires a long dynamic scan that lasts at least 60 min. This prolonged image acquisition time may cause patient discomfort and movement during clinical procedures. Therefore, several published studies have examined the possibility of reducing the scan time required to estimate K_i_ values. Recently, Puri et al.^10^ found that K_i_ values obtained from a short scan of 12-min using the Hawkins two-tissue compartmental model achieved similar statistical accuracy to those obtained from the 60-min dynamic scan. However, their short scan method did not study in the lumbar spine, which is also an important site for ^18^F-NaF PET imaging for bone turnover assessment. Peters et al.^11^ examined a correlation between the 30-min K_i_ value and the mean standardized uptake value (SUV) at 30-min and 60-min, offering clinically relevant dynamic parameters for the diagnosis of pseudarthrosis. However, a 60-min dynamic imaging was not performed in their study because the long scan time was impractical in patients with significant back pain. Therefore, the comparison of K_i_ between 30-min and 60-min dynamic scans was not investigated. Siddique et al.^12^ estimated the K_i_ from a series of 4-min static scan, simulated from a dynamic scan acquired in a time window 30–60 min after injection. While they found that bone plasma clearance obtained from a single static image can provide an accurate and robust estimate of K_i_ comparable to the conventional Hawkins 60-min dynamic scan, this approach requires taking several venous blood samples to estimate the arterial input function (AIF).

Investigations regarding the shortened scan time of ^18^F-NaF K_i_–Patlak especially in CKD patients undergoing hemodialysis, as published in previously studies remain limited. It is worthwhile to explore efforts to shorten the scan time for K_i_ value estimation for noninvasive biomarker-based bone turnover prediction, focusing on the anterior iliac crest (the site of bone biopsy) and the lumbar spine. Therefore, this study investigated the reduction of scan time for K_i_ value assessment at both the lumbar spine and anterior iliac crests for dynamic ^18^F-NaF PET/CT imaging for patients with CKD undergoing hemodialysis instead of a routine 60-min or 90-min dynamic scan. Furthermore, this study aimed to validate that a shorter scan time can provide K_i_ equivalent to that obtained from a prolonged scan.

## Material and methods

### Subjects

This study was approved by the Institutional Review Board of the Faculty of Medicine, Chulalongkorn University (IRB No.0625/65, COA No. 1644/2022). Twenty-seven Thai patients with ESRD on hemodialysis were recruited in this prospective study between December 2021 and August 2023. The patient group consisted of 15 females and 12 males, with a mean age of 54.8 ± 10.4 years, body weight of 57.6 ± 12.0 kg, body mass index of 23.0 ± 5.1 kg/m^2^, and injected activity of ^18^F-NaF of 132 ± 41.3 MBq. Patient’s demographic data included in this study are presented in Table 1. Thirteen patients underwent two scans, and one patient underwent three scans in order to evaluate the treatment responses, with a minimum of 3 months between each PET/CT examination. The remaining patients underwent one scan each. Therefore, the total number of scans to be analyzed in this study was 42. The inclusion criteria were as follows: patients who had undergone hemodialysis treatment for > 6 months with stable thrice-weekly hemodialysis, and those who exhibited a low bone mineral density T-score at the total hip or femoral neck, less than ˗1. Patients with a history of post orthopedic surgeries or those with significant bone disorders that affect the lumbar vertebrae (L1–L4) and iliac crests, current diagnosis of active malignancy, current or history of corticosteroids use for > 6 months, and history of organ transplantation were excluded from this study. Biochemical testing for bone turnover biomarkers (BTMs), such as parathyroid hormone (PTH), bone-specific alkaline phosphatase (BsAP), and tartrate-resistant acid phosphatase isoform 5b (TRAP5b), was performed before or after hemodialysis sessions.

**Table 1:** Patient’s demographic data included in this study.

Characteristics	Mean ± SD (*n* = 27)
Female	15 (55.5%)
Age (years)	54.8 ± 10.4
Height (cm)	158.5 ± 8.3
Weight (kg)	57.6 ± 12.0
Body mass index (kg/m^2^)	23.0 ± 5.1
Dialysis vintage (years)	6.3 ± 3.2
Ex-smoker	2 (7.4%)
Alcohol drinking	0
Coronary artery disease	2 (7.4%)
Heart failure	1 (3.7%)
Stroke	1 (3.7%)
Peripheral artery disease	1 (3.7%)
Hypertension	15 (55.5%)
Diabetes mellitus	5 (18.5%)
Parathyroidectomy	2 (7.4%)
Menopause	8 (53.3% of female)
History of fracture	1 (3.7%)
Osteoporosis	13 (48.1%)
Osteopenia	14 (52.9%)

### Dynamic PET/CT acquisition

All patients underwent ^18^F-NaF PET/CT dynamic scans using a digital PET/CT Siemens Biograph Vision 600 scanner (Siemens Healthineers, Erlangen, Germany) at the Division of Nuclear Medicine, King Chulalongkorn Memorial Hospital. This PET/CT system has an axial field of view (FOV) of 26.3 cm for lutetium oxyorthhosilicate (LSO) crystals and is combined with a 64-slice CT scanner. In this study, two acquisition protocols were used: 90- and 60-min dynamic scan protocols. Eight scans were performed using continuous bed motion (CBM) acquisition on a whole-body 90-min dynamic scan, and 34 scans were performed using the 60-min dynamic scan. For the 90-min scan, a 6-min dynamic single-bed list-mode PET acquisition centered at the heart region, in which the FOV encompasses the arch of the aorta to the second lumbar vertebra (L2) for extracting the image-derived input function (IDIF), started immediately after intravenous injection of ^18^F-NaF. A subsequent set of 16 multi-pass dynamic whole-body PET scans covering areas from the base of the skull to the upper thigh using CBM of approximately 5 min each with table speed 2.4 mm/s was then acquired. A 4-min pause was added between these two steps before starting dynamic whole-body scans following the Siemens multiparametric PET suite protocol^13^. For the 60-min scan, a dynamic single-bed PET acquisition with FOV encompassing the mid-twelfth thoracic vertebra (T12) to the pelvis was acquired, followed by a subsequent routine whole-body static scan of approximately 10 min.

List-mode data were reconstructed and re-binned into 42 timeframes for 90-min scans (12 × 5 s, 6 × 10 s, 8 × 30 s, 4-min pause, 16 × 300 s), and 35 timeframes for 60-min scans (12 × 5 s, 6 × 10 s, 6 × 30 s, 11 × 300 s). All images were reconstructed using the ordered subset expectation maximization (OSEM) algorithm with a Gauss filter and the point spread function (PSF) TrueX (Gauss and all pass filter) algorithm with four iterations and five subsets and a matrix size of 220 × 220. Low-dose CT protocol for attenuation correction (120 kV, CARE Dose 4D automatic tube-current modulation, pitch 0.8, rotation time 0.5 s, 3.0 mm slice thickness, 780 mm axial FOV) encompassing the base of the skull to the upper thigh was used to acquired CT images for both protocols.

### Kinetic modeling and data analysis

PMOD (version 4.002; PMOD Technologies LLC, Switzerland) was used to draw the volume of interest (VOI) on dynamic images for data analysis and K_i_–Patlak kinetic model fitting. Motion correction of the dynamic PET images was performed by averaging across the early time-frame series to create a reference for performing rigid body transform across all frames of the dynamic PET. Two input data were required for K_i_ quantification using the Patlak analysis method, that is, IDIF and bone time–activity curve (BTAC) obtained from the dynamic images.

#### Image-derived input function (IDIF)

In this study, a noninvasive method, IDIF, was used for alternative arterial blood sampling^14,15^. The VOIs were drawn within the abdominal aorta at the level of the twelfth thoracic spine (T12) to the second lumbar vertebra (L2) of the dynamic PET image using 3D cuboid with dimensions between 3 × 3 × 5 mm to 5 × 5 × 25 mm in the early phase time frames (approximate twelve 5-s), which can be used to visualize ^18^F-NaF concentration within the aorta and allows an accurate quantification of IDIF for calculating K_i_^16^. To maintain the consistency of the measurement, the same VOI was duplicated for all PET time frames. Thus, all individual IDIFs derived from 42 studies were generated at 0–90 min for eight studies and 0–60 min for thirty-four studies.

To obtain the IDIF for the short scan method, the time frames of 0–15, 0–30, 0–45, and 0–60 min were selected from 90-min PET dynamic data in PMOD to generate IDIF_15min_, IDIF_30min_, IDIF_45min_, and IDIF_60min,_ respectively_._ Similarly, for 60-min dynamic data, IDIF_15min_, IDIF_30min_, and IDIF_45min_ were generated from PET images at 0–15, 0–30, and 0–45 min, respectively.

#### Bone time–activity curves (BTACs)

**Figure 1:**
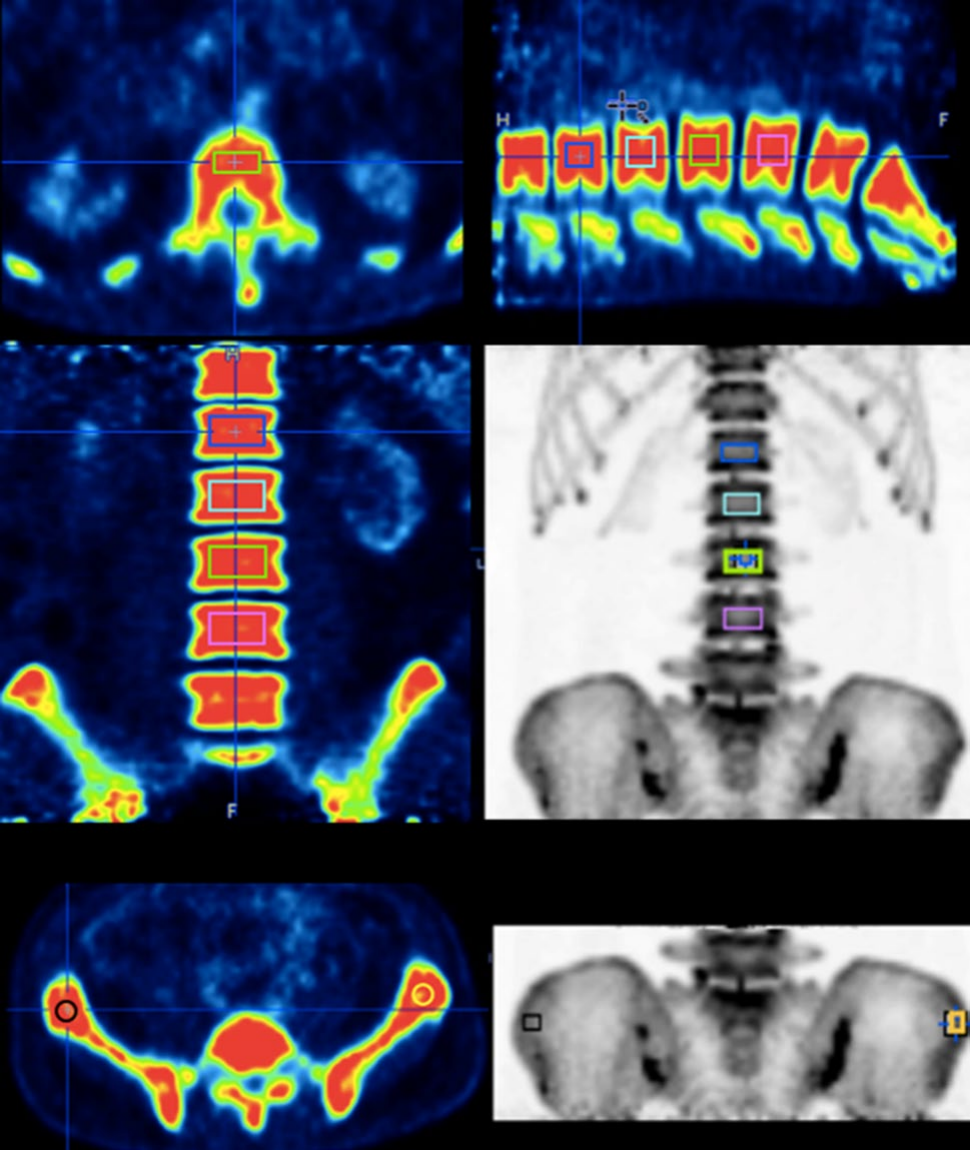
Illustration of a volume of interest drawn at four lumbar vertebrae (L1–L4) and both iliac crest regions for measuring activity concentration.

In this study, BTAC were obtained by measuring the activity concentration of ^18^F-NaF within the VOI regions of lumbar vertebrae and both iliac crests. To ensure consistency in contouring across participants, for the lumbar spine, the 3D cuboidal VOIs sized approximately 1–4 cm^3^ were placed within the middle of each vertebral body L1 to L4, avoiding the end plates and the disk space, as these could influence the activity measurement. For both iliac crests, the 3D spherical VOIs were defined by contouring on the anterior iliac crest at approximately the same region as the bone biopsy, with the VOI size ranging from 0.1–0.3 cm^3^. The contouring locations of all VOIs were then verified by a nuclear medicine physician. Illustration of the lumbar vertebrae and iliac crest VOIs contouring are shown in Fig. 1. For bone VOIs, six BTACs were generated from either 60-min or 90-min PET dynamic images from each scan. BTAC_15min_, BTAC_30min_, and BTAC_45min_ were generated from the 60-min dynamic data at 0–15, 0–30, and 0–45 min, respectively, whereas BTAC_15min_, BTAC_30min_, BTAC_45min_, and BTAC_60min_ were generated from the 90-min dynamic data at 0–15, 0–30, 0–45, and 0–60 min, respectively, for the shortened dynamic scan method.

#### K_i_–Patlak analysis

All BTACs and IDIFs were generated using the PMOD view module and subsequently transferred to the PMOD kinetic module to estimate the regional K_i_ values at the lumbar spine and iliac crest VOIs. For the Patlak analysis*,* K_i_ was obtained from Eq. (1) as follows^17^:

1 $${C}_{Bone}\left(T\right)={K}_{i}{\int }_{0}^{T}{C}_{Blood}\left(t\right)dt+ {V}_{0}{C}_{Blood}\left(T\right)$$

where ${C}_{Bone}(T)$ represents the total amount of ^18^F-NaF in bone VOI at time T after injection, ${C}_{Blood}(t)$ is the total amount of ^18^F-NaF in whole blood at each time point (t), K_i_ is the plasma clearance that describes the rate of entry into the peripheral compartment, and V_0_ is the volume of distribution of ^18^F-NaF, which shows the proportion of bone to blood activity in the bone VOI^17^. ${K}_{i}\underset{0}{\overset{T}{\int }}{C}_{Blood}\left(t\right)dt$ represents the amount of ^18^F-NaF trapped in the bound bone pool, whereas ${V}_{0}{C}_{Blood}(T)$ represents the amount of ^18^F-NaF in the unbound bone pool. Equation (1) can be divided by ${C}_{Blood}(T)$ to obtain a linear relationship with a slope equal to K_i_ and an intercept equal to V_0_. The K_i_ slope corresponds to the ^18^F-NaF trapping ratio in bone VOI.

For the initial parameter fitting in this study, the Patlak plot model allows fitting a linear regression to data starting at t*, which can be specified manually from 0 to 60 with a default weighting constant. The results are the regression slope (K_i_) and intercept (V). A maximum value of 10% was set as the error criterion.

### Statistical analysis

The Wilcoxon test was used to assess the statistical significance of the difference between K_i_–Patlak values in the lumbar spine and both anterior iliac crests. *P*-values of 0.05 were used to denote statistical significance for this test. Correlations between K_i_–Patlak values obtained from the four lumbar vertebrae and both iliac crests were assessed using Spearman’s rank correlation test (r_s_). The intraclass correlation coefficient (ICC) was used to evaluate the agreement between K_i_–Patlak derived from a shortened scan time and K_i_–Patlak derived from a long routine scan time.

### Ethics approval

This study was performed in line with the principles of the Declaration of Helsinki. Approval was granted by the Ethics Committee of the Faculty of Medicine, Chulalongkorn University (IRB No.0625/65, COA No. 1644/2022).

### Consent to participate

Informed consent was obtained from all individual participants included in this study.

## Results

### Routine K_i_–Patlak values

The mean routine K_i_–Patlak values from 42 dynamic ^18^F-NaF PET/CT scans were 0.068 ± 0.022 mL/min/mL in the lumbar spine and 0.069 ± 0.027 mL/min/mL in both anterior iliac crests as shown in Table 2. No significant difference in the means of routine K_i_–Patlak values was observed between the two regions for the 60 min routine scan time (*p* = 0.866). According to bone turnover-based classification established by Aaltonen et al.^17^, the specific cutoff values with a threshold between 0.038 and 0.055 mL/min/mL were used to distinguish low turnover from non-low turnover, and high turnover from non-high turnover in the lumbar spine and anterior iliac crest regions. In this study, 33 scans (78.6%) were classified as high and non-low turnovers, 4 scans (9.5%) were classified as normal turnover, and 5 scans (11.9%) were classified as low and non-high turnovers for both regions. The K_i_–Patlak values in the lumbar spine were compared with those in both anterior iliac crests using Spearman’s correlation test, which showed a significant correlation (r_s_ = 0.772, *p* < 0.001). Additionally, a strong correlation was observed between the average K_i_–Patlak values in the L1-L4 and each lumbar region, and between K_i_–Patlak values in both anterior iliac crests and each iliac crest region.

**Table 2:** Comparison of the K_i_–Patlak values (mL/min/mL) between the lumbar spine (L1-L4) and iliac crest regions for routine and shortened scan times.

n = 42	Lumbar spine	Both iliac crests	*p-value*
15 min	0.077 ± 0.018 (0.035–0.128)	0.071 ± 0.030 (0.035–0.161)	*0.017*
30 min	0.073 ± 0.016 (0.038–0.109)	0.071 ± 0.028 (0.034–0.158)	0.179
45 min	0.070 ± 0.019 (0.030–0.106)	0.070 ± 0.029 (0.022–0.163)	0.970
60 min (Routine scan time)	0.068 ± 0.022 (0.038–0.122)	0.069 ± 0.027 (0.025–0.145)	0.866

Data are presented as means ± standard deviations (ranges).

Level of significance = *p* < 0.05.

### Routine K_i_–Patlak values and biochemical markers

A weak correlation was observed between BsAP and K_i_–Patlak values in both anterior iliac crests (r_s_ = 0.459, *p* = 0.002). No correlation was observed between TRAP5b and K_i_–Patlak values in lumbar spine (r_s_ = 0.094, *p* = 0.553) and both anterior iliac crests (r_s_ = 0.284, *p* = 0.068) or between BsAP and K_i_–Patlak values in the lumbar spine (r_s_ = 0.079, *p* = 0.621).

### Shortened scan K_i_–Patlak and routine K_i_–Patlak values

**Figure 2:**
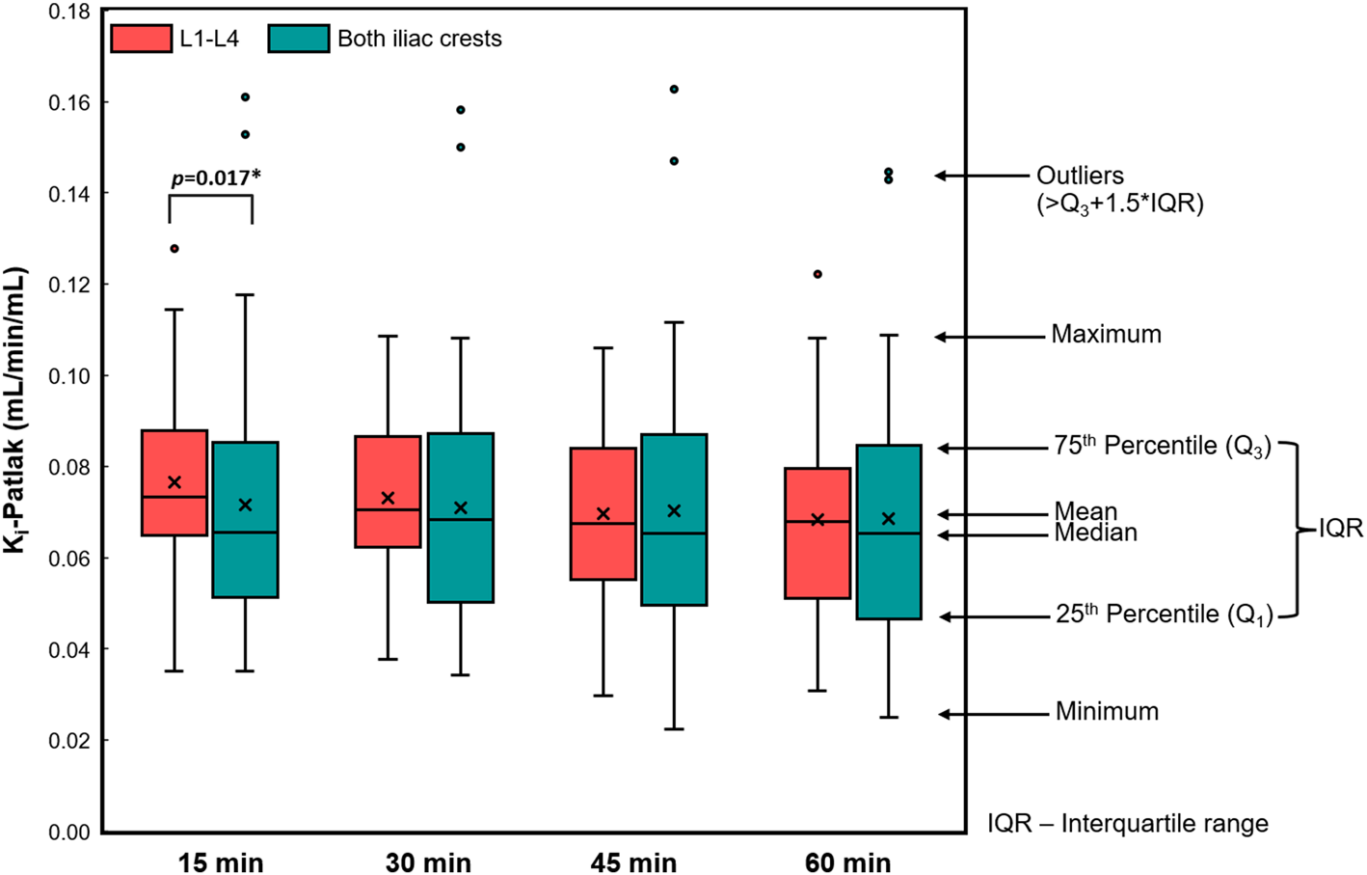
Box-and-whisker plot showing the distribution of K_i_–Patlak values calculated from shortened scan times of 15, 30, 45, and 60 min at the lumbar spine and both anterior iliac crests obtained from 42 dynamic ^18^F-NaF PET/CT scans.

**Figure 3:**
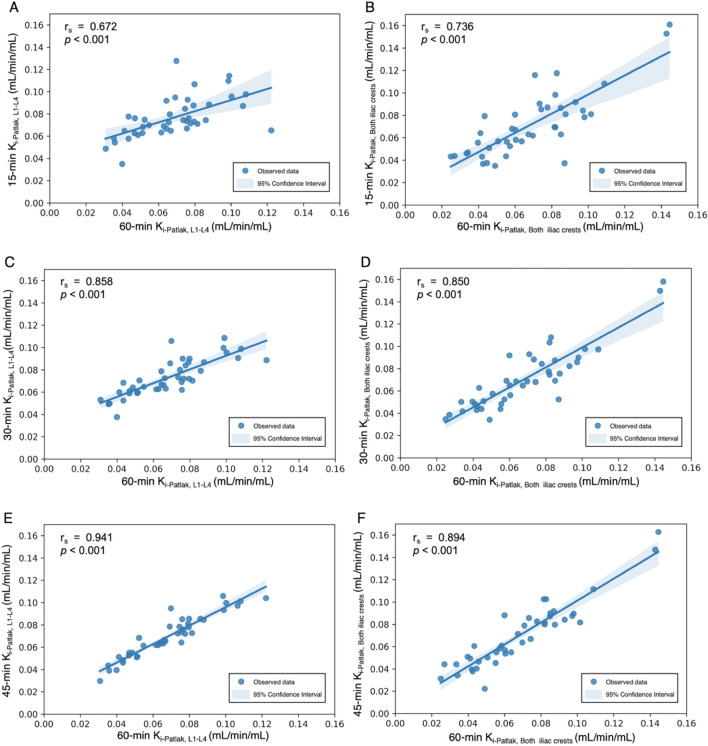
Correlation between shortened scan and long scan K_i_–Patlak values in the lumbar spine and iliac crest regions (*n* = 42). The light blue shaded area surrounding the correlation line represents the 95% confidence interval.

**Figure 4:**
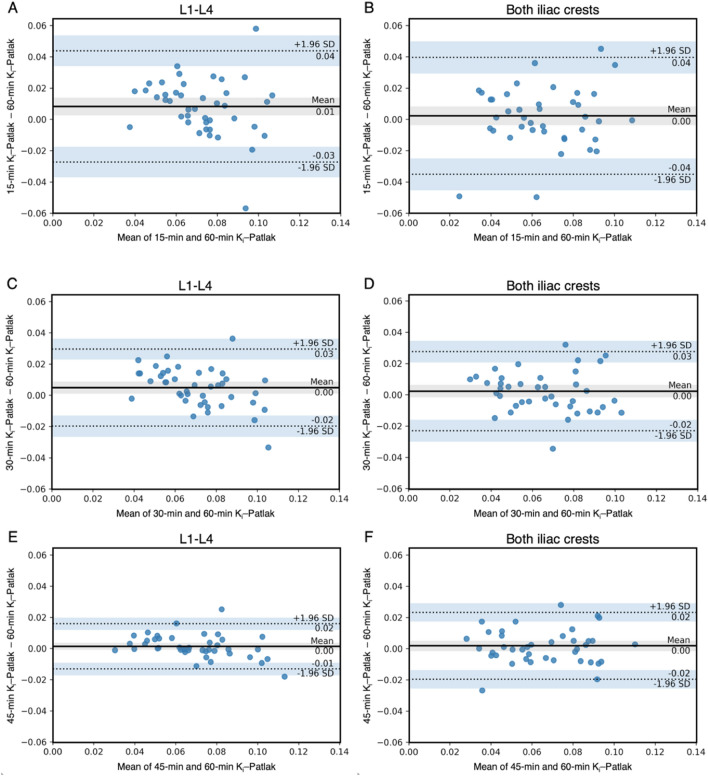
Bland–Altman plots demonstrating the relationship between the shortened scan and long scan K_i_–Patlak values in the lumbar spine and iliac crest regions. Limits of agreement are indicated by dotted lines with 95% confidence interval (light blue regions), while bias is represented by a solid line with 95% confidence interval (gray region).

The mean K_i_–Patlak values from the shortened scan time in L1–L4 and both anterior iliac crests are presented in Table 2. No significant differences in the means of 30-min and 45-min K_i_–Patlak values were observed between the two regions (*p* = 0.179 and *p* = 0.970). Figure 2 shows the distribution of K_i_–Patlak values calculated from shortened scan times at 15, 30, 45, and 60 min at the lumbar spine and both anterior iliac crests obtained from 42 dynamic ^18^F-NaF PET/CT scans. The correlations of the shortened scan K_i_–Patlak values in the lumbar region and at both iliac crests are shown in Fig. 3A–F. A strong correlation was observed between a 60-min K_i_–Patlak and the shortened scan K_i_–Patlak at 15 min (r_s_ = 0.672, *p* < 0.001), 30 min (r_s_ = 0.858, *p* < 0.001), and 45 min (r_s_ = 0.941, *p* < 0.001) in the lumbar region. This correlation was also significant between a 60-min K_i_–Patlak and the shortened scan K_i_–Patlak at 15 min (r_s_ = 0.736, *p* < 0.001), 30 min (r_s_ = 0.850, *p* < 0.001), and 45 min (r_s_ = 0.894, *p* < 0.001) at the anterior iliac crest region. Figure 4A–4F show the Bland–Altman plot of the mean difference between the shortened scan and long scan K_i_–Patlak values in the lumbar spine and anterior iliac crest regions (Table 3).

**Table 3:** Spearman correlation (r_s_) and intraclass correlation coefficient (ICC) for K_i_–Patlak from shortened and routine long scan times in the lumbar spine and anterior iliac crests.

Shortened – Routine	r_s_, *p-value*	ICC (95% CI)
Lumbar spine (L1–L4)		
15 min	0.672, < 0.001	0.547 (0.275–0.734)
30 min	0.858, < 0.001	0.764 (0.584–0.869)
45 min	0.941, < 0.001	0.934 (0.881–0.964)
Both iliac crests		
15 min	0.736, < 0.001	0.785 (0.634–0.878)
30 min	0.850, < 0.001	0.888 (0.803–0.938)
45 min	0.894, < 0.001	0.925 (0.865–0.959)

CI, confidence interval.

**Figure 5:**
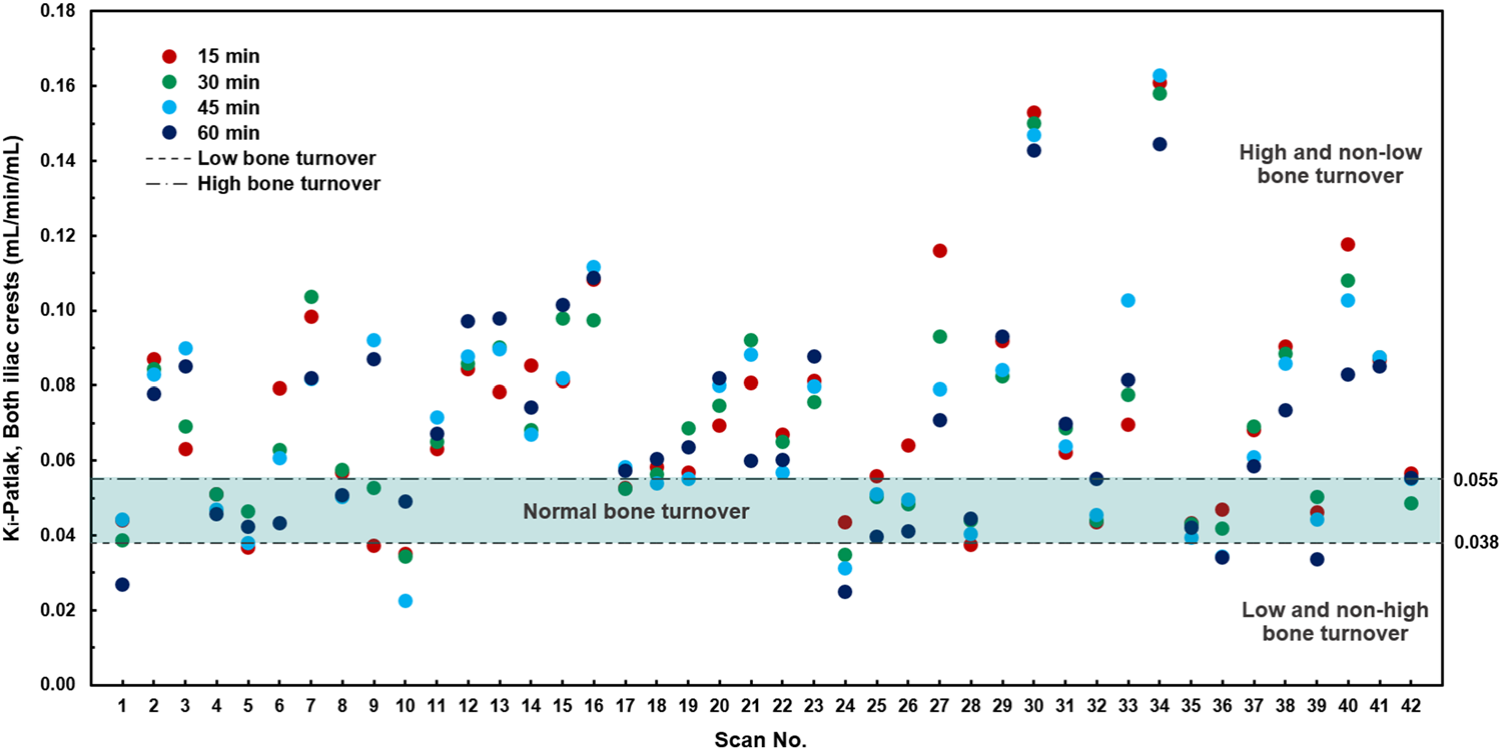
Scatter plot illustrating shortened and routine scans K_i_–Patlak values in the anterior iliac crest regions across low (< 0.038 mL/min/mL), normal (0.038–0.055 mL/min/mL), and high bone turnover (> 0.055 mL/min/mL) categories from 42 dynamic ^18^F-NaF PET/CT scans.

No significant difference of routine dynamic scan K_i_–Patlak values was found among different bone turnover categories in the two regions as shown in Table 4. These findings correspond with the K_i_–Patlak values obtained from the shortened scan time, as shown in Fig. 5. When verifying the K_i_–Patlak values obtained from shortened scan with routine 60-min scan, the results presented in Table 4 and the consistency observed with K_i_–Patlak from the shortened scan in the anterior iliac crests, as illustrated in Fig. 5, demonstrate that the selected cutoff values for bone turnover-based classification hold promise for distinguishing different turnover categories in both regions. However, further validation studies are required to extend these finding to provide a more comprehensive understanding of the utility and accuracy of the proposed threshold in clinical applications in CKD patients with hemodialysis.

**Table 4:** K_i_–Patlak values in the lumbar spine (L1–L4) and at the both iliac crest regions according to bone turnover-based classification ^18^.

n = 42	High* and non-low bone turnover (n = 33)	Normal bone turnover (n = 4)	Low** and non-high bone turnover (n = 5)
K_i_–Patlak, L1-L4 (mL/min/mL)	0.076 ± 0.018 (0.040–0.122)	0.045 ± 0.003 (0.041–0.049)	0.038 ± 0.007 (0.031–0.047)
K_i_–Patlak, Both iliac crests (mL/min/mL)	0.077 ± 0.025 (0.042–0.145)	0.044 ± 0.005 (0.040–0.051)	0.033 ± 0.008 (0.025–0.046)
*p-*value	0.908	0.465	0.225

Data are presented as means ± standard deviations (ranges).

*Cutoff > 0.055 mL/min/mL.

**Cutoff < 0.038 mL/min/mL.

Level of significance = *p* < 0.05.

## Discussion

The K_i_–Patlak derived from dynamic ^18^F-NaF PET/CT in patients on hemodialysis shows a significant correlation with histomorphometric parameters obtained from bone biopsies^5,6^. This highlights the potential of using bone plasma clearance K_i_–Patlak of ^18^F-NaF PET/CT as an alternative noninvasive biomarker method to evaluate bone turnover changes in ROD. Typically, kinetic modeling of ^18^F-NaF requires a long scan time with consequent patient discomfort, limiting its use in clinical implementation. This study investigated the feasibility of K_i_–Patlak derived from a shortened scan time for dynamic ^18^F-NaF PET/CT to predict bone turnover in CKD patients undergoing hemodialysis, compared to those obtained from a traditional long dynamic scan in the lumbar spine (L1–L4) and both anterior iliac crests. The results showed that K_i_–Patlak values in the L1–L4 and anterior iliac crests derived from the 30-min scan were reliable, supported by the high correlation with K_i_–Patlak values at the 60-min scan and the high ICC for K_i_–Patlak values at 30- and 60-min scans in both regions. The observed correlation can be attributed to the kinetics of ^18^F-NaF, indicating that high-contrast bone imaging can be achieved as early as 30–45 min after injection. Therefore, a 30-min K_i_–Patlak can effectively capture bone turnover changes, aligning with the prediction of both 60-min and 90-min K_i_–Patlak. Based on the findings in this study, the results were consistent with previous literatures, particularly the study of Fuglø et al.^3^ who highlighted the potential of a 30-min dynamic ^18^F-NaF PET/CT scan, spanning from the 5th lumbar vertebra to the proximal femur, for assessing bone turnover. They demonstrated a strong correlation between K_i_ values obtained through five non-invasive methods and those derived from the arterial input function method. Peters et al.^11^ found a strong correlation between the 30-min K_i_ value and the mean SUV at 30- and 60-min, showing clinically significant dynamic parameters for diagnosing pseudarthrosis. Furthermore, Siddique et al.^12^ indicated the feasibility of estimating the K_i_ values from a 4-min static ^18^F-NaF PET scan acquired between 30–60 min post-injection. In contrast, it is noteworthy that an uptake period less than or equal 15 min may not provide sufficient data for quantitative methods in clinical interpretation.

There was a slightly lower K_i_–Patlak in anterior iliac crests compared to lumbar spine. Additionally, the distribution of K_i_–Patlak in the anterior iliac crests for all scan times exhibited a wider range than that in the lumbar spine. These results align with the findings of Puri et al.^19^ who mentioned that K_i_ values are influenced by bone blood flow, and lower bone blood flow in the hip is a significant factor for K_i_ quantification. Nevertheless, no significant differences were observed in the mean routine K_i_–Patlak and shortened scan K_i_–Patlak values between the lumbar spine and anterior iliac crest regions, except at 15 min.

Regarding biochemical markers, a weak correlation was observed between BsAP and K_i_–Patlak values, particularly at the anterior iliac crest. Additionally, no significant correlation was found between TRAP5b and either K_i_–Patlak values or between BsAP and K_i_–Patlak values in the lumbar spine. It is noted that circulating bone turnover markers, such as BsAP and TRAP5b, may reflect overall skeletal metabolism, including cortical and trabecular bones. In contrast, K_i_–Patlak derived from PET scans represents regional metabolism within specific skeletal regions. However, 28 out of 42 scans (66.67%) can provide the BsAP and TRAP5b biomarker testing results agreed with routine K_i_–Patlak values from both regions, indicating the potential benefits of using biochemical testing as a noninvasive validation method for bone turnover prediction.

When measuring K_i_ using various methods with clinical potential, the derivation of an individual input function (IF) for each participant is required^16^. In this study, the abdominal aorta region was used to derive the IF because several prior studies have indicated that the IDIF can be extracted from the aorta rather than the left ventricle^14,20,21^. However, partial volume corrections were not performed in this study primarily because all participants were adults, and the diameter of the abdominal aorta vessel significantly exceeded the resolution of the digital PET scanner^21^. In line with this decision, Lodge et al.^22^ suggested that the partial volume effect (PVE) is unlikely to introduce significant error in aortic VOI measurements, and PVE can be neglected for large vessels.

The significance of this study lies in the extensive number of scans conducted in Thai CKD patients, and the assessment of bone metabolic flux at the lumbar vertebrae and both anterior iliac crests, corresponding to the biopsy site, using dynamic scanning in both regions. Most previous studies of bone tracer kinetics employed the dynamic scanning to estimate K_i_ at selected skeletal site, typically either the lumbar spine or hip, as the information was often constrained by the field of view of conventional PET/CT scanner^3,9–12^. Despite the primary limitation of this study being the lack of bone histomorphometric parameters obtained from bone biopsy, attributed to patient discomfort and challenges in operation room setup, additional investigations were conducted to further explore correlations between biochemical testing (TRAP5b and BsAP) and K_i_–Patlak values^23^. The clinical implications of these findings are that shortened scan K_i_–Patlak may aid treatment decisions and decrease the need for invasive bone biopsies in patients with CKD. The challenges in establishing a normal value range from histomorphometric biopsy results may arise from several factors, such as race, age, and sex, which can introduce variations^18^. Healthy volunteers are required to assess the normal range of K_i_ in future studies. Furthermore, in this study, we did not investigate sex differences in K_i_–Patlak values in bone turnover prediction. This limitation is acknowledged, and further research is suggested to explore potential sex-dependent variations in bone turnover rate estimation. This would allow for a more comprehensive analysis and understanding of the factors influencing bone metabolism.

## Conclusions

This study shows that the proposed shortened scan time for performing ^18^F-NaF PET dynamic measurements of K_i_–Patlak bone metabolic flux at the lumbar spine and iliac crest regions can evaluate bone turnover in patients with CKD equivalent to that derived from a long dynamic scan. The implications of these findings are significant, potentially offering guidance for alternative treatment decisions, reducing the impact of patient movement during scans, and minimizing the need for invasive bone biopsies.

## Data Availability

The datasets generated during and/or analyzed during the current study are available from the corresponding author on reasonable request.

## Abbreviations

AIF: Arterial input function
BsAP: Bone-specific alkaline phosphatase
BTAC: Bone time-activity curve
BTMs: Bone turnover biomarkers
CBM: Continuous bed motion
CKD: Chronic kidney disease
ESRD: End-stage renal disease
^18^F-NaF: Fluorine–18–labeled sodium fluoride
IDIF: Image-derived input function
K_i_: Bone metabolic flux
PTH: Parathyroid hormone
PVE: Partial volume effect
ROD: Renal osteodystrophy
SUV: Standardized uptake value
TMV: Turnover, mineralization, and volume
TRAP5b: Tartrate-resistant acid phosphatase isoform 5b

## References

[1] KDIGO 2017 Clinical practice guideline update for the diagnosis, evaluation, prevention, and treatment of chronic kidney disease-mineral and bone disorder (CKD-MBD). *Kidney Int. Suppl.* **7**, 1–59 (2017). 10.1016/j.kisu.2017.04.001 PMC6340919 30675420

[2] Hsu CY, Chen LR, Chen KH (2020). Osteoporosis in patients with chronic kidney diseases: A systemic review. Int. J. Mol. Sci..

[3] Fuglø D, Drachmann ALP, Heltø KMM, Marner L, Hansen D (2023). Bone turnover in patients with chronic kidney disease stage 5D and healthy controls - a quantitative [^18^F]fluoride PET study. Mol. Imaging Biol..

[4] Puri T (2023). Utility of a simplified [^18^F] sodium fluoride PET imaging method to quantify bone metabolic flux for a wide range of clinical applications. Front. Endocrinol. (Lausanne).

[5] Aaltonen L (2020). Correlation between ^18^F-sodium fluoride positron emission tomography and bone histomorphometry in dialysis patients. Bone..

[6] Messa C (1993). Bone metabolic activity measured with positron emission tomography and [^18^F]fluoride ion in renal osteodystrophy: correlation with bone histomorphometry. J. Clin. Endocrinol. Metab..

[7] Czernin J, Satyamurthy N, Schiepers C (2010). Molecular mechanisms of bone ^18^F-NaF deposition. J. Nucl. Med..

[8] Blake GM, Siddique M, Frost ML, Moore AEB, Fogelman I (2012). Quantitative PET imaging using (^18^)F sodium fluoride in the assessment of metabolic bone diseases and the monitoring of their response to therapy. PET Clin..

[9] Vrist MH (2022). Bone turnover, mineralization, and volume estimated by ^18^F-sodium fluoride PET/CT and biomarkers in chronic kidney disease: Mineral and bone disorder compared with bone biopsy. Am. J. Nephrol..

[10] Puri T, Siddique MM, Frost ML, Moore AEB, Blake GM (2021). A short dynamic scan method of measuring bone metabolic flux using [^18^F]NaF PET. Tomography..

[11] Peters MJ, Wierts R, Jutten EMC, Halders SGEA, Willems PCPH, Brans B (2015). Evaluation of a short dynamic ^18^F-fluoride PET/CT scanning method to assess bone metabolic activity in spinal orthopedics. Ann. Nucl. Med..

[12] Siddique M (2012). Estimation of regional bone metabolism from whole-body ^18^F-fluoride PET static images. Eur. J. Nucl. Med. Mol. Imaging..

[13] Fahrni G, Karakatsanis NA, Domenicantonio GD, Garibotto V, Zaidi H (2019). Does whole-body Patlak (18)F-FDG PET imaging improve lesion detectability in clinical oncology?. Eur Radiol..

[14] Cook GJR, Lodge MA, Marsden PK, Dynes A, Fogelman I (1999). Non-invasive assessment of skeletal kinetics using fluorine-18 fluoride positron emission tomography: Evaluation of image and population-derived arterial input functions. Eur. J. Nucl. Med..

[15] Khamwan K (2023). Dynamic ^18^F-FDG-PET kinetic parameters for epileptogenic zone localization in drug-resistant epilepsy. Front. Phys..

[16] Puri T, Frost ML, Moore AEB, Cook GJR, Blake GM (2023). Input function and modeling for determining bone metabolic flux using [^18^F] sodium fluoride PET imaging: A step-by-step guide. Med. Phys..

[17] Assiri R, Knapp K, Fulford J, Chen J (2022). Correlation of the quantitative methods for the measurement of bone uptake and plasma clearance of 18F-NaF using positron emission tomography. Systematic review and meta-analysis. Eur. J. Radiol..

[18] Aaltonen L (2021). Bone histomorphometry and ^18^F-sodium fluoride positron emission tomography imaging: comparison between only bone turnover-based and unified TMV-based classification of renal osteodystrophy. Calcif. Tissue Int..

[19] Puri T (2013). Differences in regional bone metabolism at the spine and hip: A quantitative study using (^18^)F-fluoride positron emission tomography. Osteoporos. Int..

[20] Puri T (2011). Validation of new image-derived arterial input functions at the aorta using ^18^F-fluoride positron emission tomography. Nucl. Med. Commun..

[21] van der Weerdt AP, Klein LJ, Boellaard R, Visser CA, Visser FC, Lammertsma AA (2001). Image-derived input functions for determination of MRGlu in cardiac (^18^)F-FDG PET scans. J. Nucl. Med..

[22] Lodge MA, Lesniak W, Gorin MA, Pienta KJ, Rowe SP, Pomper MG (2021). Measurement of PET quantitative bias in vivo. J. Nucl. Med..

[23] Jørgensen HS (2022). Diagnostic accuracy of noninvasive bone turnover markers in renal osteodystrophy. Am. J. Kidney Dis..

